# Evolution of the Critically Endangered Green Sawfish *Pristis zijsron* (Rhinopristiformes, Pristidae), Inferred from the Whole Mitochondrial Genome

**DOI:** 10.3390/genes14112052

**Published:** 2023-11-08

**Authors:** Chen Wang, Peiyuan Ye, Richard Pillans, Xiao Chen, Junjie Wang, Pierre Feutry

**Affiliations:** 1 State Key Laboratory of Marine Environmental Science, College of Ocean and Earth Sciences, Xiamen University, Xiamen 361000, China; wangchen2971@163.com; 2 College of Marine Sciences, South China Agricultural University, Guangzhou 510642, China; cuiluoshi1998@163.com (P.Y.); chenxiao@scau.edu.cn (X.C.); 3 CSIRO Environment, Boggo Road, Dutton Park, QLD 4102, Australia; richard.pillans@csiro.au; 4 Guangzhou Key Laboratory of Subtropical Biodiversity and Biomonitoring, School of Life Sciences, South China Normal University, Guangzhou 510631, China; 5 CSIRO Environment, Castray Esplanade, Hobart, TAS 7000, Australia

**Keywords:** *Pristis zijsron*, mitochondrial genome, critically endangered, phylogenetic analysis, divergence time

## Abstract

The green sawfish *Pristis zijsron* (Bleeker, 1851), a species of sawfish in the family Pristidae (Rhinopristiformes), mainly inhabits the Indo-West Pacific region. In this study, the complete mitochondrial genome of the critically endangered green sawfish is first described. The length of the genome is 16,804 bp, with a nucleotide composition of 32.0% A, 24.8% C, 13.1% G, and 30.0% T. It contains 37 genes in the typical gene order of fish. Two start (GTG and ATG) and two stop (TAG and TAA/T-) codons are found in the thirteen protein-coding genes. The 22 tRNA genes range from 67 bp (tRNA-*Ser*) to 75 bp (tRNA-*Leu*). The ratio of nonsynonymous substitution (Ka) and synonymous substitution (Ks) indicates that the family Pristidae are suffering a purifying selection. The reconstruction of Bayesian inference and the maximum likelihood phylogenetic tree show the same topological structure, and the family Pristidae is a monophyletic group with strong posterior probability. *Pristis zijsron* and *P. pectinata* form a sister group in the terminal clade. And the divergence time of Rhinopristiformes show that *P. zijsron* and *P. pectinata* diverged as two separate species in about Paleogene 31.53 Mya. Complete mitochondrial genomes of all five sawfishes have been published and phylogenetic relationships have been analyzed. The results of our study will provide base molecular information for subsequent research (e.g., distribution, conservation, phylogenetics, etc.) on this endangered group.

## 1. Introduction

Chondrichthyans (sharks, rays, and chimeras) are the most evolutionary distinct radiation of vertebrates, and the entire clade has been determined as one of the first major marine fish lineages facing extinction risk [1,2]. The genomic architecture of chondrichthyans may be closer to the ancestral vertebrate condition compared with scleractinians, but there is limited available research [3,4]. It is noteworthy that the life history traits (e.g., slow growth, low fecundity in long gestation boxes, etc.) of cartilaginous fishes make them more susceptible to overfishing problems compared with bony fishes [5]. Rhinopristiformes is an order of rays that have shark-like features, which is presumably a combination of Rhinobatiformes and Pristiformes, two nominal orders in which taxa had previously been placed, including at least 71 species in 12 genera [6,7]. This order is known by a few common names, such as guitarfish, giant guitarfish, sawfish, etc. The populations of many Rhinopristiformes species are declining in heavily fished areas, and are facing a high risk of extinction [8,9]. Therefore, more attention should be paid to this group.

Although sawfishes are similar to sharks, they are actually rays. The green sawfish *Pristis zijsron* (Bleeker, 1851) mainly inhabits the shallow waters of coastal marine, mangrove, or estuarine habitats of the Indo-West Pacific region [10,11]. And it is the largest sawfish species of family Pristidae, reaching an impressive length of up to 24 feet. In Australia, the distribution of green sawfish is from the east coast of the central Pacific Ocean, around the northern continent, to Shark Bay in the south-west to the eastern Indian Ocean, but the population in north-eastern Australia has now declined dramatically, and may even be extinct along much of the east coast of Australia [12,13,14]. On the contrary, the species is still regularly found in western Australia. Due to habitat destruction and modification, populations of sawfishes have been impacted worldwide [15,16]. The International Union for the Conservation of Nature and Natural Resources (IUCN) Red List of Threatened Species declared that the family Pristidae is the most threatened elasmobranch in the world because of their declining population numbers, and all international commercial trade of five sawfishes was prohibited by the Convention on International Trade in Endangered Species of Flora and Fauna (CITES) Appendix I [17,18]. In addition, four *Pristis* (dwarf sawfish *P. clavate*, smalltooth sawfish *P. pectinata*, largetooth sawfish *P. pristis* and green sawfish *P. zijsron*) are currently assessed as “Critically Endangered” under criteria A2cd [14,19,20,21], because of being highly vulnerable to entanglement in gillnets and demersal trawl nets. Although it has never been quantified, it is believed that *P. zijsron* is experiencing drastic reduction in population size across its range and no longer appears to be common anywhere in its range. In the last 50 years, it has no longer appeared to be common anywhere within its range, and it is possibly extinct in some countries, for example, China, Thailand, Singapore, etc. [14].

In general, mitochondria are thought to have originated from Alphaproteobacteria, through the process of endosymbiosis existing in eukaryotic cells [22,23]. The mitochondrial genome synthesizes proteins autonomously and participates in different physiological functions in organisms [24]. Fish mitochondrial DNA is a circular, covalently closed double-stranded DNA molecule of 15–20 kilobases (kb) in length, and is considered to have an evolutionary origin independent of nuclear DNA, generally comprising 13 protein-coding genes (PCGs), 22 transfer RNA genes (tRNAs), 2 ribosomal RNA genes (rRNAs), an open reading frame (O_L_), and a control region (CR) [25,26]. Moreover, the mitochondrial genome is often used as a reliable marker to study the origin and phylogenetic relationship of species due to its fast evolution rate, maternal inheritance, and small molecular weight. Although accurately identifying sawfish species is a difficult task, the mitochondrial DNA may provide a powerful tool for scientists who are not expert taxonomists [27,28]. Molecular data will greatly contribute to other related studies of sawfishes. Recently, Haque et al. used DNA barcoding (*COI* gene) to verify the recording of green sawfish *P. zijsron* in Bangladeshi waters [29]. And Aschliman et al. used 12 H-strand PCGs to explore batoid evolution [4].

Here, we add the first whole mitochondrial genomic sequence for *P. zijsron*, and use it to predict the phylogenetic position of the sawfish species. Furthermore, we investigate the divergence time of Rhinopristiformes using a dataset (12 H-strand PCGs and rRNAs) in conjunction with fossil records. It is believed that the study will provide important molecular data for subsequent studies on the distribution, conservation, and evolution of sawfish species.

## 2. Materials and Methods

### 2.1. Sample Collection and Sequence Analysis

The sample (fin clip) of *P. zijsron* was collected from a specimen captured and released at Cape Keraudren, Western Australia, Australia, on 14 April 2008. The specimen was a male measuring 260 cm total length. The tissue sample was stored at CSIRO, Castray Esplanade, Hobart, Tasmania, Australia, with voucher no. 1034 G. The genomic DNA was extracted from the fin clip by the standard phenol-chloroform procedure [30]. LA-PCR reactions were performed using the Takara^TM^ LA-Taq DNA polymerase kit. The PCR primers and PCR reaction conditions closely followed a previous study [31].

Sequence data were generated and analyzed to map the complete mitochondrial genome using the Lasergene soft package [32]. The species was identified by morphology and fragments of *COI* and 16S rRNA gene downloaded from National Center of Biotechnology Information (NCBI) (https://www.ncbi.nlm.nih.gov/genbank/, accessed on 1 September 2023). The mitochondrial genome was firstly annotated by MITOS2 Web Server [33]. The 22 tRNA genes’ secondary structures were determined by tRNAscan-SE Search Server v2.0 [34], then further confirmed with default search mode in ARWEN v1.2 [35]. Annotation and accurate boundary determination of PCGs and rRNAs genes were aligned and manually compared with the other released reference genomes of Pristidae species using DNAman v9.0 [36]. Tandem Repeats Finder was used to find tandem repeats in the non-coding regions (NCR), then secondary structures were identified by Mfold v3.5. [37].

**Figure 1:**
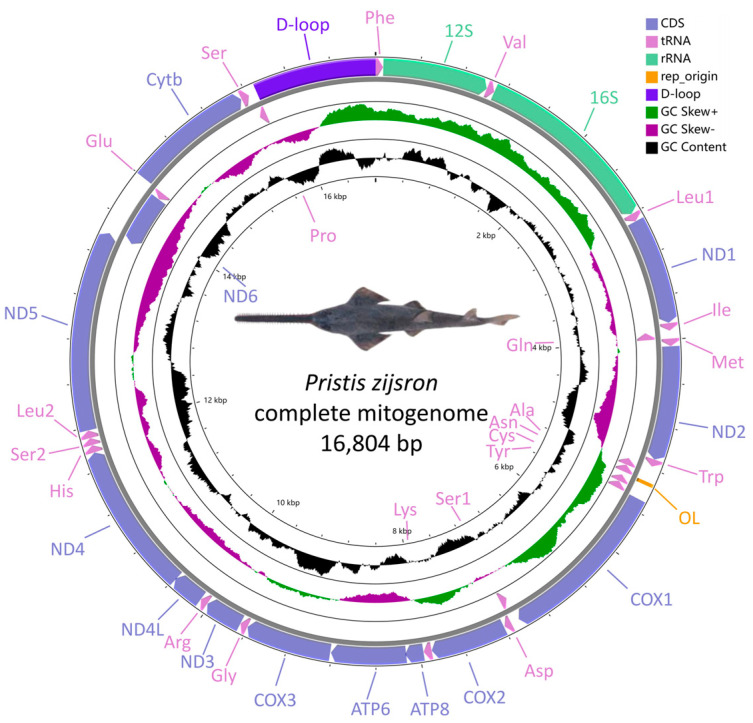
Circular representation of the mitochondrial genome of *Pristis zijsron*.

The *P. zijsron* mitochondrial genome was drawn into a full circular genome using CGView Server v1.0 (Figure 1) [38]. The base compositions, codon usage, and relative synonymous codon usage (RSCU) values were calculated in MEGA X [39], then plotted using ggblot2 by Rstudio. Nucleotide diversity (Pi), non-synonymous substitutions (Ka), and synonymous substitution (Ks) mutation rate ratios of the PCGs of the Pristidae mitochondrial genome were calculated with Dnasp v6 [40]. The strand asymmetry was assessed using the formulas provided as below: AT skew was calculated as (A − T)/(A + T), and GC skew was calculated as (G − C)/(G + C) [41].

### 2.2. Phylogenetic Analysis

Phylogenetic analyses were performed using the new mitochondrial genome and other Rhinopristiformes species with complete mitochondrial genomes downloaded from GenBank (Table 1). *Callorhinchus milii* (NC_014285) and *Tor putitora* (NC_021755) were chosen as outgroups. The DNA sequences of 12 H-strand PCGs and rRNAs genes were firstly aligned using the MAFFT program, and were then re-aligned with MACSE v2 software [42,43]. And the alignments of H-strand PCGs (excluding the start, stop, and the third codon) were divided into the first and the second codon positions. Gblocks was employed to eliminate ambiguously aligned segments from the rRNA alignments [44]. The final dataset comprised the above segments after concatenation using Phylosuite v1.2.3 [45].

The three segments of the dataset were pre-defined as partitions and verified by PartitionFinder v2.1.1 [59], and the ModelFinder program was used to select the best-fit partition model [60]. The GTR+F+I+G4 model was selected as the best one and three partitions were defined: (1) two rRNA genes; (2) the first; and (3) the second codons of 12 PCGs. In addition, phylogenetic reconstruction was performed using the Bayesian analyses (BI) and Maximum Likelihood (ML) methods. ML analysis was inferred under 1,000,000 ultrafast bootstraps with an approximate Bayes test using IQ-TREE v1.6.2 [61]. Based on the above partition model, Bayesian inference was conducted using MrBayes 3.2.6 software, under partition model (2 parallel runs, 1 × 10^6^ generations), in which the initial 25% of sampled data were discarded as burn-in [62]. Subsequently, the tree files were employed for visualization and annotation by the Figtree program.

### 2.3. Divergence Time Analysis

Divergence times were calculated within a Bayesian framework using the aforementioned dataset and BEAST version 2.5 [63]. The uncorrelated lognormal relaxed clock, GTR substitution model, gamma site heterogeneity model, and the Yule process tree prior were selected to estimate differentiation time. Then, the divergence time analysis used three time calibration points derived from the TimeTree online [64]: 55.3 million years ago (Mya) for the common ancestor of *P. clavata* and *P. pristis*; 85.6 Mya for the common ancestor of the genera *Rhina* and *Pristis*; and 159.2 Mya for the common ancestor of Rhinopristiformes.

The chains of 1 × 10^8^ samples were run for the Markov chain Monte Carlo (MCMC) analysis, and 10% of all samples had burn-in using TreeAnnotator software. Tracer v1.7.1 was used to analyze the results [65]. Mean ages, along with 95% highest posterior density (HPD) intervals, posterior probabilities, and substitution rates, were computed for every node. The Figtree program was used to visualize the tree file.

## 3. Results and Discussion

### 3.1. Mitochondrial Genome Structure and Organization

The *P. zijsron* mitochondrial genome (GenBank accession No. MH005927) is 16,804 bp in length. The gene content of *P. zijsron* was similar to the typical fish mitochondrial genome, including 37 typical regions, 13 protein-coding (PCGs), 22 transfer RNA (tRNAs), 2 ribosomal RNA (rRNAs) genes, and 1 non-coding control region, with the typical gene order and transcriptional orientation of most vertebrate mitochondrial genomes (Figure 1 and Table 2). All genes were encoded on the heavy-strand (H-strand), except the *ND6* and eight tRNA genes (Gln, Ala, Asn, Cys, Thr, Ser, Glu and Pro) located on the light-strand (L-strand). The size and location of each gene were also similar to most fish mitochondrial genomes.

There were 39 base pairs (bp) of short intergenic regions situated at 15 gene junctions, along with 24 bp overlaps identified at 6 gene junctions. Among all these intergenic regions, the shortest overlap was 10 bp in length (found between tRNA-*Lys* and *ATP8*), while the longest spacer was 6 bp in length (found between *ATP8* and *ATP6*). In addition, the *P. zijsron* mitochondrial genome’s O_L_ was positioned between the tRNA-*Asn* and tRNA-*Cys* gene, spanning a length of 33 bp. Furthermore, three tRNA genes (Ile, Gln, Met) and five tRNA genes (Trp, Ala, Asn, Cys, Tyr) were observed to form clusters typically referred to as the “IQM” and “WANCY” regions.

**Figure 2:**
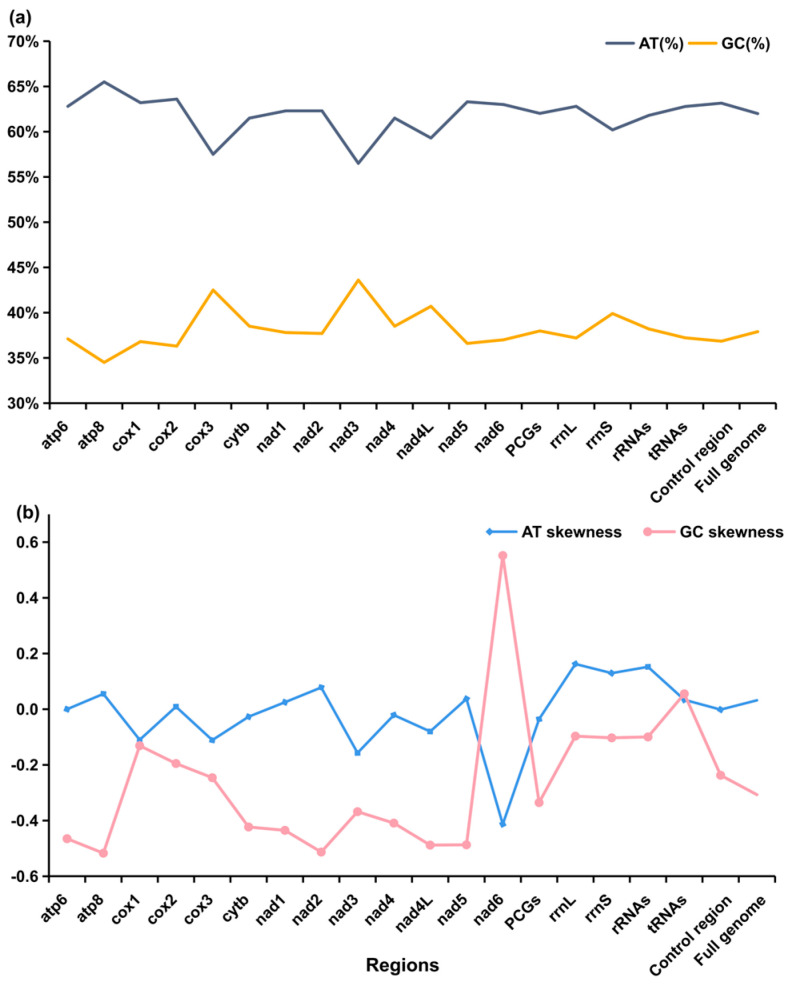
Visual representation showing the base composition (**a**) and skewness (**b**) in the mitochondrial genome of *Pristis zijsron*.

The nucleotide composition was 32.0% adenine (A), 24.8% cytosine (C), 13.1% guanine (G), and 30.0% thymine (T), respectively. The base skew was also measured. The AT-skews of the entire mitochondrial genome of *P. zijsron* were positive (indicating an excess of A content over T), while the GC-skews were negative, indicating a higher G content than C content. Additionally, the AT-skews of 12 H-strand PCGs genes were more positive than GC-skews. The results indicate the A content was higher than the T content, and the C content was higher than the G content for the *P. zijsron* mitochondrial genome (Figure 2). And the AT content of the complete *P. zijsron* mitochondrial genome was 62.0%, similar to four other published Pristidae mitochondrial genomes (Table 1) [31,47,48,49].

### 3.2. Protein Coding Genes and Codon Usage

The lengths of 13 PCGs range from 297 bp (*ND4L* gene) to 1839 bp (*ND5* gene). The *COI* gene starts with a GTG codon, which is normal in fish mitochondrial genomes [66]. The remaining protein genes start with the conventional ATG codon. Except for the *ND6* gene terminating with the TAG codon, the other genes ended with TAA/T- codons. The 12 H-strand PCGs were T-rich, whereas the *ND6* gene encoded on the L-strand was A-rich (Figure 2).

**Figure 3:**
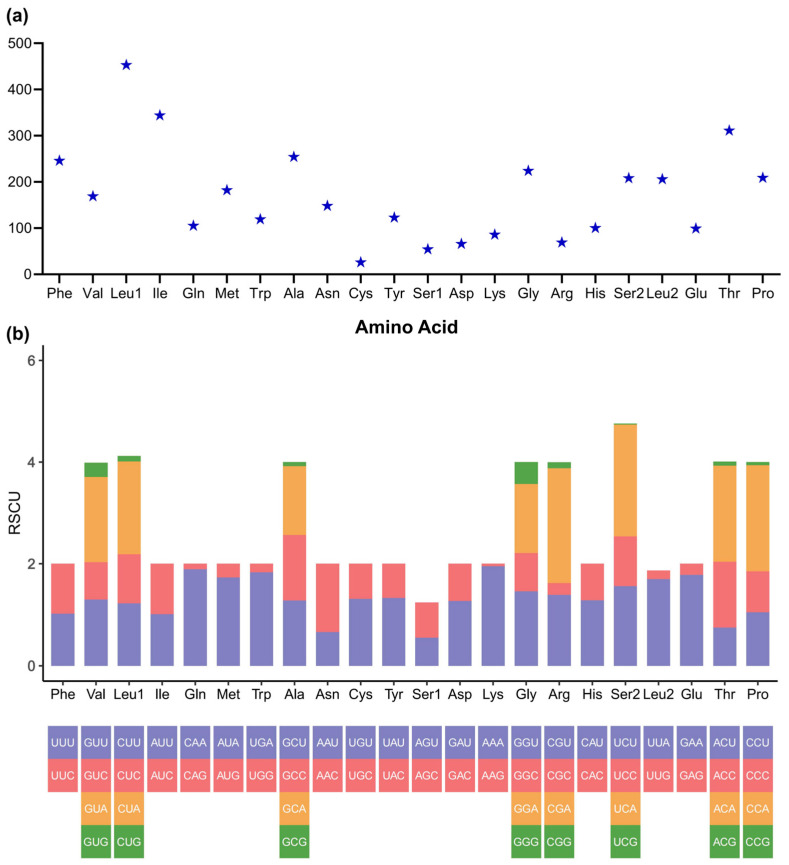
The codon distribution (**a**) and relative synonymous codon usage (**b**) in *Pristis zijsron* mitochondrial genome.

The most frequent amino acids in the PCGs of *P. zijsron* mitochondrial genome proteins were Leucine (Leu) and I-isoleucine (Ile), similar to other published Pristidae mitochondrial sequences (Figure 3a). Moreover, Cysteine (Cys) was the least frequently utilized amino acid in the mitochondrial sequence of *P. zijsron*. Simultaneously, the relative synonymous codon usage (RSCU) values of the *P. zijsron* mitochondrial genome were computed and are presented in Figure 3b. The findings revealed a bias toward A and T in the third codons. The length, RSCU, skewness, and AT content of PCGs in the *P. zijsron* mitochondrial genome were nearly identical, similar to other published Pristidae sequences.

**Figure 4:**
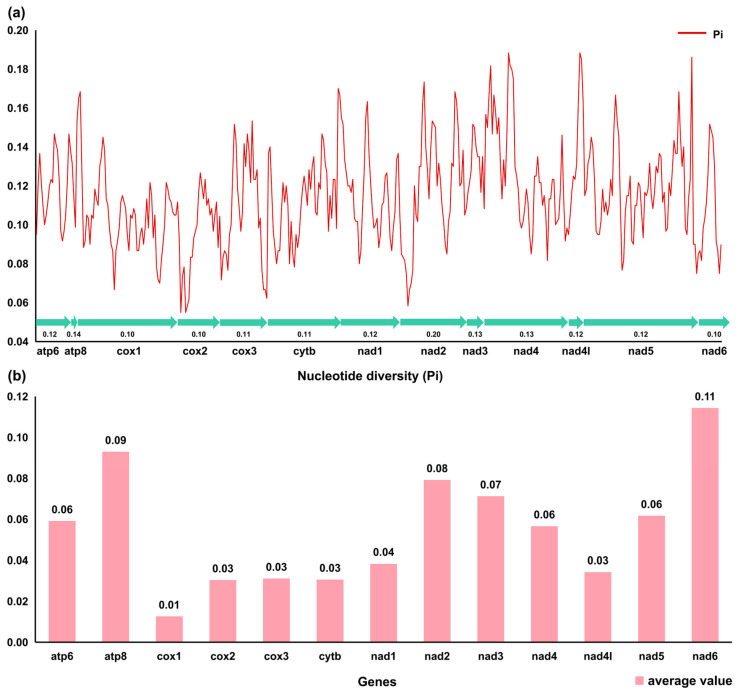
Graphical illustration showing the sliding window analysis based on 13 PCGs of Pristidae mitochondrial genomes (**a**). The red line represents the value of nucleotide diversity observed in the sliding window analysis (window size = 100 bp, step size = 25 bp). The graph displays gene names and corresponding Pi values, while the arrows indicate the length of the 13 PCGs. Additionally, it illustrates the non-synonymous (Ka) to synonymous (Ks) substitution rates for 13 PCGs within the Pristidae family (**b**).

Four species of sawfishes were grouped in pairs and their Ka/Ks were calculated, as shown in Figure 4. The Ka/Ks ratios of the 13 PCGs in 4 Pristidae mitochondrial genomes were all much less than 1. The highest average Ka/Ks ratio (0.11) was found in the *ND6* gene, and the lowest average ratio (0.01) was found in the *COI* gene. Therefore, it can be considered that the genes had undergone purifying selection, and the evolution tended to be conservative.

### 3.3. Transfer RNAs, Ribosomal RNAs, and the D-Loop

Like the typical set of tRNA genes in fish mitochondrial genomes, 22 standard tRNA genes were found, and most of them could be folded into the typical cloverleaf structure, except for tRNA-*Ser* gene lacking the entire dihydrouridine arm. It had two kinds of tRNA-*Leu* and tRNA-*Ser* (Table 2). The lengths of 22 tRNAs were varied, ranging from 67 bp (tRNA-*Ser*) to 75 bp (tRNA-*Leu*), and the AT content was larger than the GC content (Figure 2a).

The 12S rRNA (963 bp) and 16S rRNA (1695 bp) genes were located between the tRNA-*Phe* and tRNA-*Leu* genes, separated by a tRNA-*Val* gene. The two rRNAs, both encoded by the H-strand, indicated moderate nucleotide compositional bias towards AT content (Figure 2). Moreover, the AT-skew of rRNAs was strongly positive, whereas the GC-skew was slightly negative, so a higher A and C content compared with T and G content in these regions was indicated.

The non-coding regions in the *P. zijsron* mitochondrial genome include the control regions (CR), the origin of L-strand replication (O_L_), and several short intergenic spacers that range in size from 1 to 7 bp. The AT-rich CR, located between tRNA-*Pro* and tRNA-*Phe* with a length of 1110 bp, is the largest of these non-coding regions. The length of the CR is variable, and this is also the main reason for the differences in fish mitochondrial genome lengths. The CR of *P. zijsron* genomes showed positive AT-skew values. The origin of the L-strand replication sequence was identified between the tRNA-*Asn* and the tRNA-*Cys* genes. It was 33 bp in length and can be folded into a hairpin structure as a signal to initiate the replication of the L-strand.

### 3.4. Phylogenetic Inference

**Figure 5:**
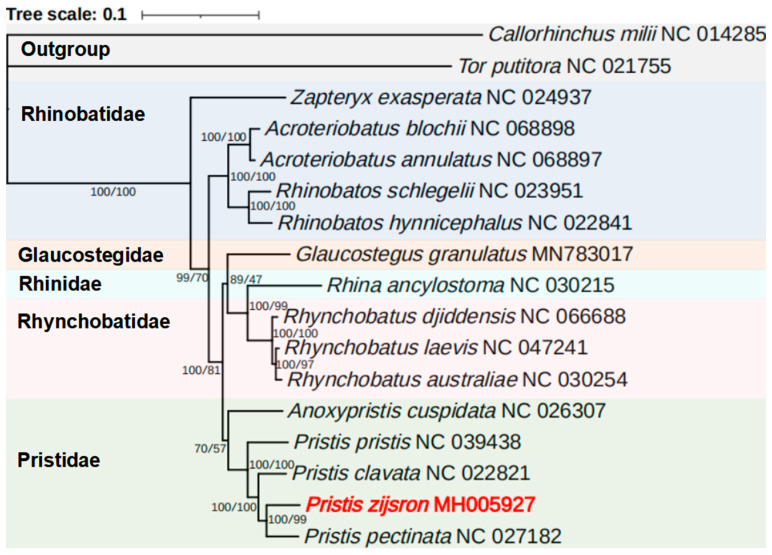
Mitogenomic phylogeny of Rhinopristiformes and two outgroups based on 12 H-strand PCGs and rRNA genes using the ML and BI methods.

There is considerable attention being paid to the evolution of critically endangered sawfishes, and a robust phylogenetic analysis is essential for accurate research. For further investigation of the phylogenetic relationships of Rhinopristiformes, phylogenetic trees were constructed to compare with published complete mitochondrial genomes and two outgroups using the BI and ML methods. The two different approaches produced identical topologies with high nodal support values (Figure 5). Consistent with the traditional taxonomy, the phylogenetic reconstruction in this study strongly supported the monophyly of five families (Glaucostegidae+ Pristidae+ Rhinidae+ Rhinobatidae+ Rhynchobatidae) of Rhinopristiformes. Family Pristidae is farther from Rhinobatidae than other Rhinopristiformes.

The phylogeny of the family Pristidae is now fully resolved. All five species are clustered together with the four *Pristis* species (*P. zijsron*, *P. pristis*, *P. clavate,* and *P. pectinata*), forming a monophyletic group. *Pristis zijsron* and *P. pectinata* form a sister group in the terminal clade, then cluster with *P. clavata* and *P. pristis*. *Anoxypristis cuspidata* is placed in the basal position of the family Pristidae. Rhinopristiformes is a large taxa including at least 78 species [11], and some new species of this group have been discovered in recent years [67,68,69,70,71,72]. Hence, the whole mitochondrial genomes of more species need to be studied to further investigate the phylogenetic relationships of this taxa. Additional mitochondrial genomes of Rhinopristiformes can be supporting evidence for morphological identification, especially for critically endangered species, and it will be useful to substantiate the conclusion in future studies.

### 3.5. Divergence Time Analysis

**Figure 6:**
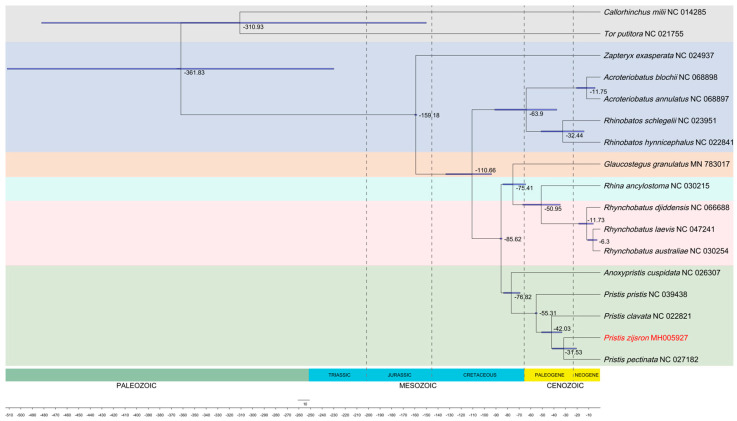
Divergence time of Rhinopristiformes and two outgroups based on 12 H-strand PCGs and rRNA genes using BEAST2 software.

Divergence time typically denotes the estimated moment when two species or organism groups diverged from a shared ancestor, initiating independent evolutionary paths. Few studies have attempted to use mitochondrial genomic data to explore the evolution of sawfishes. To provide a timeline of *P. zijsron* evolution, the divergence time estimation of Rhinopristiformes and other related species was conducted using the Bayesian approach (Figure 6). The results show that Rhinopristiformes diverged from two outgroups (*C. milii* and *T. putitora*) in the Jurassic, about 159.18 Mya. Within the order Rhinopristiformes, the family Rhinobatidae firstly diverged from other species in the Cretaceous, about 110.66 Mya. The common ancestor of the family Pristidae occurred in 76.82 Mya. *Pristis pectinata* and *P. zijsron* diverged as two separate species at about 31.53 Mya, as the most recently diverged species in TimeTree. Notably, the genus *Pristis* exhibited a divergence time dating back to 55.31 Mya, earlier than the fossil records of *P. pectinatus* (3.6–5.333 Mya) and *P. pristis* (5.333–11.608 Mya) [73,74]. Similar to a previous Batoidea study, the major groups of Rhinopristiformes are estimated to have separated during the Cretaceous period. Subsequently, from the Late Cretaceous to the Cenozoic era, different subgroups emerged within each branch.

However, it is important to note that the divergence time was estimated using temporal calibration points on TimeTree, due to the lack of sufficiently accurate fossil evidence. Estimates with calibration points may lead to biased results, so the divergence time results can only be used as a preliminary reference in this study. In future, it will be necessary to add a lot of molecular data to Rhinopristiformes species and associated fossil evidence to this group, which will be helpful in reconstructing a more detailed phylogenetic tree for estimating the divergence times. More molecular data on Chondrichthyes need to be added, and will be indispensable for subsequent morphological and evolutional studies of endangered species in Chondrichthyes.

## 4. Conclusions

We successfully identified and compared the complete mitochondrial genome of *P. zijsron* for the first time. The length of the *P. zijsron* mitochondrial genome was 16,804 bp. The ratio of Ka and Ks indicated that Pristidae underwent a purifying selection, while the *ND6* gene showed the highest Ka/Ks values (<1). Identical topologies with strong nodal support were produced by both ML and BI approaches, showing that the family Pristidae formed a monophyletic group. And the divergence time of Rhinopristiformes showed that *P. zijsron* and *P. pectinate* formed a sister group, diverging in the Paleogene, 31.53 Mya.

Additionally, there are currently no conservation methods in place for the Pristidae family. The structure and variability of the fish mitochondrial genome are still unclear. A comprehensive comparative study using full mitochondrial genomic sequences will help enhance our comprehension of the evolution profile and phylogenetic placement in the family Pristidae. It is anticipated that this study will significantly contribute to the development of research related to the resources and conservation of sawfish species.

## Figures and Tables

**Table 1:** Rhinopristiformes species information used in this study.

Family	Species	Length (bp)	AT%	GenBank ID	Reference
Glaucostegidae	*Glaucostegus granulatus*	16,547	59.8	MN783017	[46]
Pristidae	*Anoxypristis cuspidata*	17,243	61.3	NC_026307	[31]
	*Pristis clavata*	16,804	60.2	NC_022821	[47]
	*Pristis pectinata*	16,802	61.0	NC_027182	[48]
	*Pristis pristis*	16,912	60.3	NC_039438	[49]
	*Pristis zijsron*	16,804	62.0	MH005927	This study
Rhinidae	*Rhina ancylostoma*	17,217	62.3	NC_030215	[50]
Rhinobatidae	*Acroteriobatus annulatus*	16,773	59.8	NC_068897	[5]
	*Acroteriobatus blochii*	16,771	60.1	NC_068898	[5]
	*Rhinobatos hynnicephalus*	16,776	59.8	NC_022841	[51]
	*Rhinobatos schlegelii*	16,780	60.4	NC_023951	[52]
	*Zapteryx exasperata*	17,310	62.5	NC_024937	[53]
Rhynchobatidae	*Rhynchobatus australiae*	16,804	59.8	NC_030254	[54]
	*Rhynchobatus djiddensis*	16,799	60.0	NC_066688	[55]
	*Rhynchobatus laevis*	16,560	59.5	NC_047241	[56]
Outgroup	*Callorhinchus milii*	16,769	66.2	NC_014285	[57]
	*Tor putitora*	16,576	57.0	NC_021755	[58]

**Table 2:** Summary of gene features of *Pristis zijsron* mitochondrial genome.

Region	Strand	Position	Size (bp)	Start Codon	Stop Codon	Intergenic Spacer
tRNA-*Phe* (F)	H	1–69	69			0
12S rRNA	H	70–1032	963			0
tRNA-*Val* (V)	H	1033–1105	73			0
16S rRNA	H	1106–2799	1695			0
tRNA-*Leu* (L)	H	2800–2874	75			0
*ND1*	H	2875–3849	975	ATG	TAA	2
tRNA-*Ile* (I)	H	3852–3921	70			−1
tRNA-*Gln* (Q)	L	3921–3992	72			0
tRNA-*Met* (M)	H	3993–4062	70			0
*ND2*	H	4063–5109	1047	ATG	TAA	−1
tRNA-*Trp* (W)	H	5109–5177	69			2
tRNA-*Ala* (A)	L	5180–5248	69			0
tRNA-*Asn* (N)	L	5249–5321	73			0
O_L_	-	5322–5354	33			0
tRNA-*Cys* (C)	L	5355–5423	69			0
tRNA-*Tyr* (Y)	L	5424–5493	70			1
*COI*	H	5495–7051	1557	GTG	TAA	4
tRNA-*Ser* (S)	L	7056–7126	71			1
tRNA-*Asp* (D)	H	7128–7195	68			6
*COII*	H	7202–7892	691	ATG	T-	0
tRNA-*Lys* (K)	H	7893–7966	74			1
*ATP8*	H	7968–8135	168	ATG	TAA	−10
*ATP6*	H	8126–8809	684	ATG	TAA	6
*COIII*	H	8816–9601	786	ATG	TAA	1
tRNA-*Gly* (G)	H	9603–9672	70			0
*ND3*	H	9673–10,021	349	ATG	T-	1
tRNA-*Arg* (R)	H	10,023–10,092	70			1
*ND4L*	H	10,094–10,390	297	ATG	TAA	−7
*ND4*	H	10,384–11,764	1381	ATG	T-	0
tRNA-*His* (H)	H	11,765–11,833	69			0
tRNA-*Ser* (S)	H	11,834–11,900	67			−1
tRNA-*Leu* (L)	H	11,900–11,971	72			0
*ND5*	H	11,972–13,810	1839	ATG	TAA	−4
*ND6*	L	13,807–14,325	519	ATG	TAG	1
tRNA-*Glu* (E)	L	14,327–14,395	69			3
*Cytb*	H	14,399–15,541	1143	ATG	TAA	4
tRNA-*Thr* (T)	H	15,546–15,619	74			5
tRNA-*Pro* (P)	L	15,625–15,694	70			0
Control region	-	15,695–16,804	1110			0

## Data Availability

The datasets generated and analyzed during the current study are available from the corresponding author on reasonable request.
